# Multisource Heterogeneous Sensor Processing Meets Distribution Networks: Brief Review and Potential Directions

**DOI:** 10.3390/s25134146

**Published:** 2025-07-03

**Authors:** Junliang Wang, Ying Zhang

**Affiliations:** School of Microelectronics (School of Integrated Circuits), Nanjing University of Science and Technology, Nanjing 210094, China; junliang.wang@njust.edu.cn

**Keywords:** multisource heterogeneous sensor, data fusion processing, distribution network assessment, extreme conditions

## Abstract

The progressive proliferation of sensor deployment in distribution networks (DNs), propelled by the dual drivers of power automation and ubiquitous IoT infrastructure development, has precipitated exponential growth in real-time data generated by multisource heterogeneous (MSH) sensors within multilayer grid architectures. This phenomenon presents dual implications: large-scale datasets offer an enhanced foundation for reliability assessment and dispatch planning in DNs; the dramatic escalation in data volume imposes demands on the computational precision and response speed of traditional evaluation approaches. The identification of critical influencing factors under extreme operating conditions, coupled with dynamic assessment and prediction of DN reliability through MSH data approaches, has emerged as a pressing challenge to address. Through a brief analysis of existing technologies and algorithms, this article reviews the technological development of MSH data analysis in DNs. By integrating the stability advantages of conventional approaches in practice with the computational adaptability of artificial intelligence, this article focuses on discussing key approaches for MSH data processing and assessment. Based on the characteristics of DN data, e.g., diverse sources, heterogeneous structures, and complex correlations, this article proposes several practical future directions. It is expected to provide insights for practitioners in power systems and sensor data processing that offer technical inspirations for intelligent, reliable, and stable next-generation DN construction.

## 1. Introduction

The reliability and stability of distribution network (DN) operations are increasingly compromised by both natural phenomena and anthropogenic factors. It has become crucial to comprehensively and accurately assess the operational status of DN sensors, coupled with predictive analytics to evaluate future trends in active DNs [1,2]. The large-scale integration of distributed power sources, electric vehicles, and heterogeneous energy storage systems has gradually transformed the structure and configuration of DNs, giving rise to novel distribution frameworks compliant with next-generation power system requirements. Simultaneously, the proliferation of advanced energy storage technologies and flexible transmission systems has resulted in increasingly complex voltage levels within power grids, more intricate network configurations, and a greater diversity of operational methods. These changes pose substantial challenges to maintaining the safe and efficient operation of DNs.

As the terminal component of power systems, DNs bear critical responsibility for delivering electrical energy to end consumers. Their secure and stable operation is essential for maintaining the overall reliability of the power system. Industry statistics reveal that over 80% of consumer power interruptions originate from DN failures. Climatic variations and extreme weather events exacerbate the inherent uncertainties of renewable energy integration, while posing substantial risks of catastrophic equipment damage during severe meteorological conditions, thereby threatening the stability of DNs.

Meteorological hazards impacting grid stability may be categorized into two categories: direct (e.g., weather- and climate-related phenomena) and indirect (e.g., secondary and derivative meteorological disasters). Principal threats include typhoon systems, ice accretion events, seismic activities, fluvial inundations, and wildfire incidents. The increasing frequency of extreme weather events in recent years has precipitated critical challenges to power system stability. Low-probability high-consequence events may induce catastrophic equipment degradation within a short time while complicating the restoration processes of DNs, which can result in extensive and protracted service interruptions. The repercussions span from quotidian operational disruptions and industrial production stoppages to grave societal impacts, including human casualties, socio-political repercussions, and impediments to regional economic progression.

Modern DNs are characterized by integration nodes for renewable generation and heterogeneous loads, which may result in complex topologies and extensive coverage with distinct regional characteristics [3]. This highlights the necessity for real-time situational awareness in the face of uncertain conditions, such as environmental changes, distributed generation volatility, and unforeseen disaster events. Moreover, the cohabitation of aging infrastructure with modern installations, along with weak fault causality, intricate mechanisms, and significant randomness in DNs, renders conventional monitoring approaches inadequate for real-time status awareness.

**Figure 1:**
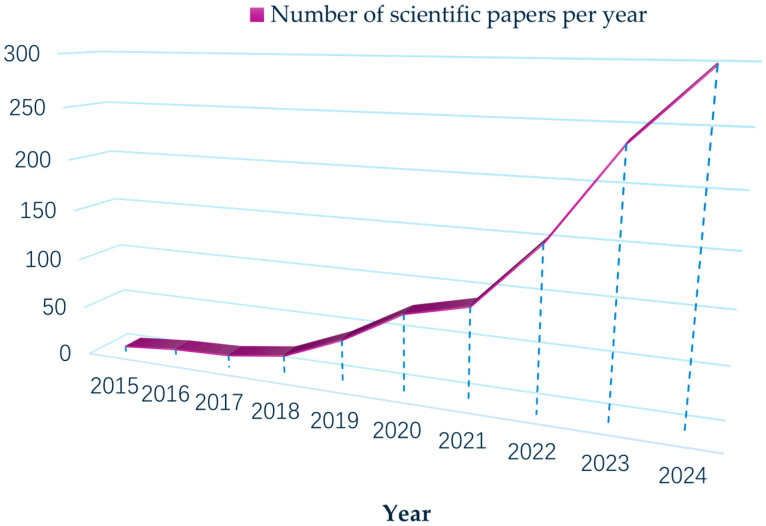
Number of scientific papers per year using multisource data processing and distribution networks simultaneously (source: Web of Science).

The relevant review of existing DN technologies focuses on grid-integrated photovoltaic systems [4], service restoration solutions [5], issues with distorted and weak power quality [6], minimizing power losses [7], and incipient fault detection [8]. There are relatively few reviews on multisource heterogeneous (MSH) data processing, and there has been no correlation review in areas such as temporary data preprocessing, MSH data fusion, or system state evaluation. This paper aims to supplement the review of such sensor data processing issues, which focuses on MSH data processing technologies for regional DNs that contain temporal data preprocessing, MSH data fusion, and system state evaluation, as quantified in Figure 1 through the number of scientific publications from 2015 to 2024. A comprehensive operational assessment requires not only DN operation data but also meteorological and hydrological information from the relevant regions. The multidimensional analysis is especially significant for effectively establishing renewable energy sources, which often come with inherent uncertainties. On the basis of investigating existing methods, we present the relevant MSH analysis methods and some conceptual frameworks for future DN system architecture. This review paper provides foundational insight for the processing and evaluation of regional DNs, particularly under extreme conditions. Finally, aiming at guiding technological evolution, this paper further outlines promising future research directions in sensor data processing.

## 2. Multisource Data for Distribution Networks

**Figure 2:**
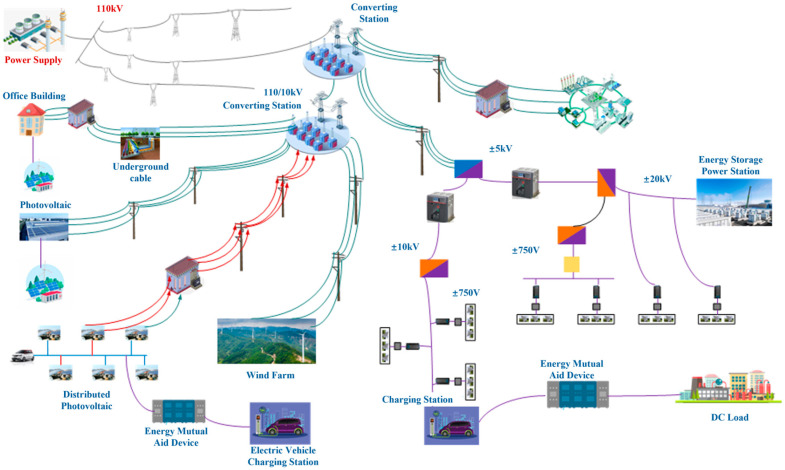
Distribution network with distributed source loads.

An example of complex DNs with distributed source loads is found in Figure 2. The MSH data sources of intelligent integration terminals for DNs can be divided into three categories, primarily electrical, meteorological, and hydrological sensors [9,10,11], as summarized in Table 1. Electrical data in DNs mainly includes voltage, current, active and reactive power, power factors, and various harmonic components. Meteorological data in DNs includes wind speed, wind direction, air temperature, air humidity, light intensity, soil temperature, soil humidity, atmospheric pressure, etc. Hydrological data in DNs mainly includes precipitation, evaporation, water level, flow, etc.

Due to various factors such as MSH sensor operation, data collection, and uploading, there are often various problems in the electrical data of DNs, such as the following:

(1) Data noise: Measurement anomalies may be caused by MSH sensor errors, electromagnetic interference, communication packet loss, etc. Due to signal pollution from multiple links and inherent nonlinear characteristics of sensors, measurement errors may also accumulate. These interference sources overlap with each other on the MSH signal transmission chains, and then form composite noise pollution that seriously distorts the true electrical characteristics.
(2) Data loss: Local data loss may be induced by offline devices, communication interruption, or storage failure. Originating from both MSH sensor abnormalities and communication failures, offline devices will create data source interruption, while storage media defects lead to historical data corruption. Its spatiotemporal distribution exhibits dynamic correlation characteristics; simultaneously, in the temporal dimension, it often presents strong correlation with MSH equipment maintenance cycles and abnormal occurrence periods.
(3) Data redundancy: This refers to repetitive collection of MSH data or periodic redundancy in time series. The periodic operation mode of massive DNs leads to repetitive features in the time dimension defined as temporal sequences, while similar measurement data of strongly correlated electrical nodes form spatial redundancy. This architectural redundancy not only occupies storage resources, but also causes feature confusion during MSH data analysis, which results in key information being diluted by a large amount of duplicate data.
(4) Inconsistent data: Time stamps from different MSH systems are not synchronized or have conflicting dimensions. The essence of this stems from a possible lack of MSH system standardization, incomplete clock synchronization of each monitoring unit leading to time reference drift, MSH sensor-design differences causing physical quantity conversion errors, semantic ambiguity of data labels causing cross-system parsing deviations, etc.
(5) High-dimensional complexity: It is challenging to analyze the correlation of massive MSH time-series data. The essence of the multidimensional spatiotemporal characteristics of the power sensors lies in the dynamic coupling of multiple physical quantities in the time dimension, and topological correlations in the MSH spatial dimension. Therefore, when the MSH operating state changes, complex nonlinear interactions occur.

For meteorological data, in terms of high-temperature environments, the performance of humidity sensors may degrade, and strong winds or airflow may cause external disturbances to the MSH sensors, accordingly affecting the stability of humidity measurement. Further, prolonged sensor use could result in component aging, which leads to increased measurement errors, inaccurate meteorological characteristic values, and even missing data.

The temperature dataset contains several outliers that should be cross-verified with contemporaneous records from corresponding periods. While specific dates exhibit temperature readings significantly exceeding regional norms, this may be due to instrument malfunctions, day–night temperature variations, and measurement errors caused by extreme weather events. Occasionally, wind direction data may have values that do not conform to the definition of wind direction, which may affect the accuracy of analytical outcomes such as wind direction frequency distribution.

## 3. Related Methods Review

**Figure 3:**
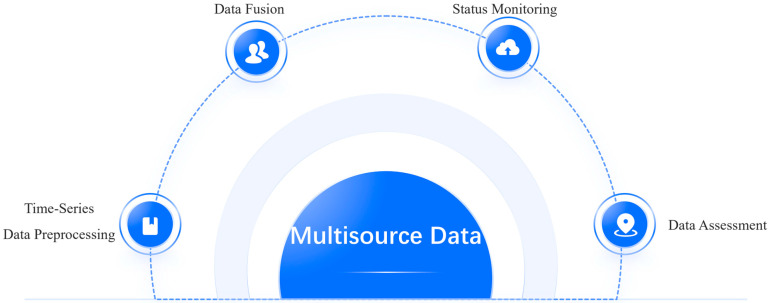
The logic and scope framework of this review.

As shown in Figure 3, multisource time-series data preprocessing, data fusion, status monitoring, and data assessment are reviewed.

### 3.1. Multisource Time-Series Data Preprocessing

The prediction of MSH and multivariable time series in DNs requires simultaneously capturing both univariate temporal dynamics and multivariate interdependencies. This methodology demonstrates application value in the field of multimodal sensor data analysis for DNs. As a typical non-stationary time series, sensor data in DNs exhibits complex dynamic characteristics driven by multiple factors, e.g., meteorological conditions, consumer behavior patterns, and social activities. Notably, such MSH data contains deterministic features, e.g., daily periodic fluctuations and long-term trend evolution, which are accompanied by local random disturbances such as equipment malfunctions and load transients. Its prediction accuracy highly depends on deep mining and fusion analysis of historical MSH data, which incorporates multidimensional DN information such as meteorological parameters, equipment status, and data types.

The high complexity of the DN operation environment will further exacerbate data quality. Factors such as MSH sensor malfunctions, storage anomalies, and communication interruptions from cyber-attacks may lead to widespread irregular missing data. This not only results in random fluctuations in time-series sampling intervals (i.e., the time decay effect diminishes the effectiveness of MSH historical information), but also significantly complicates the modeling of multivariable relationships due to the spatiotemporal heterogeneity of missing patterns across MSH parameters, all of which requires data preprocessing.

**Figure 4:**
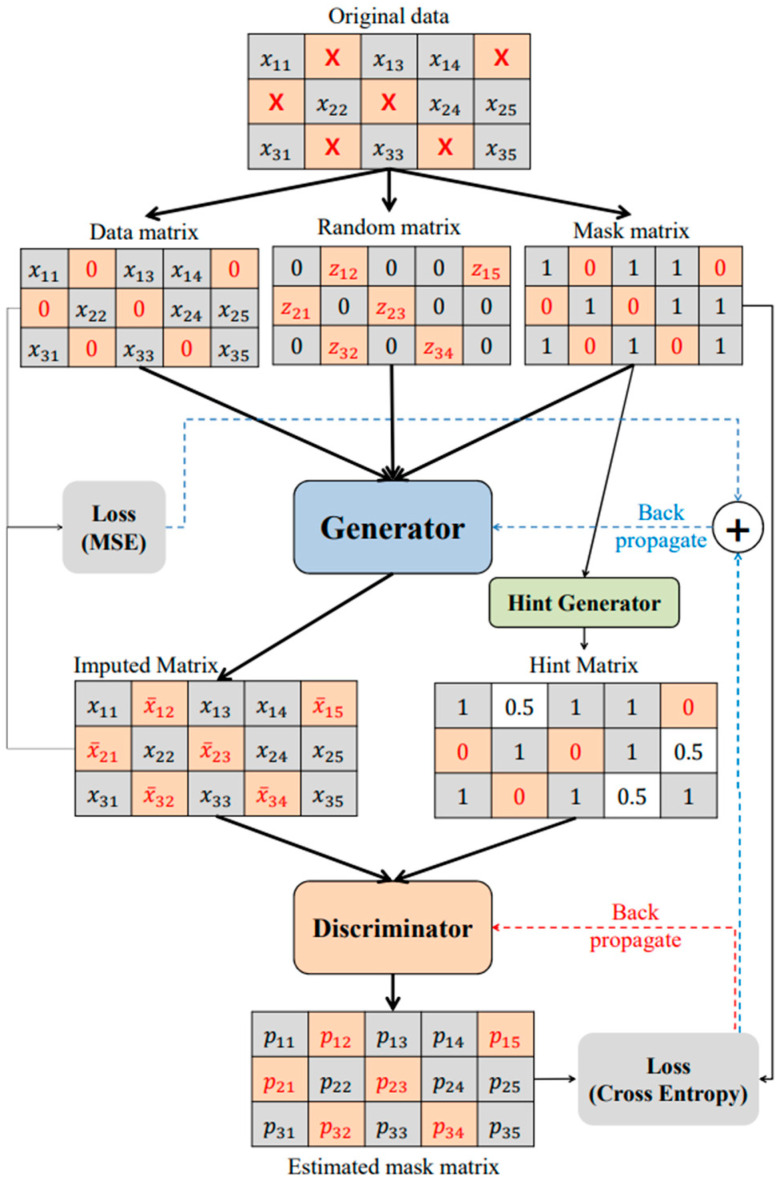
The architecture of GAIN in [16].

Data preprocessing poses significant challenges to the efficiency of predictive models and the reliability of subsequent decision-making. It may not only create analysis bias but also result in the loss of effective information. One may simply delete missing values, but this will cause changes in the distribution characteristics of the original sensor data, thereby affecting the completeness of the MSH analysis. MSH data preprocessing is primarily manifested in filling approaches, which can be divided into statistical filling, interpolation filling, filling approaches by combining multiple models, and artificial intelligence approaches. For instance, Wang et al. [12] designed a short-term power prediction approach by data cleaning and feature reconstruction to enhance MSH prediction accuracy. The authors mapped many historical samples composed of wind direction, speed, and power into a multidimensional sample, and discussed the MSH data distribution and outlier detection in different dimensions using the local density of each sample. Gupta et al. [13] established a bidirectional filling and prediction approach via a generative adversarial network (GAN), which adopted a custom generator loss function to fill missing data and a discriminator to compare the differences among observed values and filled ones. Liu et al. [14] proposed a three-step data imputation approach, which sequentially imputed these missing values according to the principle of easy to difficult, and divided them into isolated missing values, continuous missing variables, and continuous missing samples. It was achieved through MSH fragment segmentation, interpolation, and estimation approaches, as well as a stepwise extrapolation prediction model via LSTM networks. Tang et al. [15] proposed a super-resolution GAN-based interpolation to address the issue of coarse resolution in photovoltaic power generation and load consumption datasets. It provided 5 min photovoltaic and load power data from a 30 min/h temporal resolution. Yoon et al. [16] designed a GAIN algorithm for handling missing data derived from GAN. Among its requirements for data, the integrity and quality of the data were the primary factors to consider for GAIN, as reemphasized in Figure 4. High-quality data could not only enhance the training efficiency of GAIN but also promote its generalization ability.

The performance of deep learning methods such as GAN and LSTM is highly dependent on the careful selection of key parameters. For GANs, the number and width of generator and discriminator layers directly determine the model’s expressive ability and ability to learn complex data distributions. Too few layers may result in the model being unable to capture data details, while too many layers can easily lead to unstable training, mode collapse, or a significant increase in computing resources. Meanwhile, insufficient samples can exacerbate the risk of pattern collapse and limit the diversity of generated samples. In LSTM, the number of layers directly affects the depth and complexity of its modeling of long-term dependencies, but increasing the number of layers also significantly increases the training difficulty and overfitting risk. The size of the hidden layer determines the capacity of its state information. In addition, the effectiveness of LSTM in processing sequence data also depends on sufficiently long correlated sequence samples to fully learn temporal dynamic patterns. Therefore, whether it is the adversarial game balance of GAN or the temporal modeling ability of LSTM, the selection of parameters, e.g., layers, hidden units, and sample size, needs to be carefully optimized through repeated experiments and domain knowledge combined with validation strategies to achieve the best balance between model complexity, stability, generalization ability, and computational efficiency.

Based on whether the missing value filling and prediction modeling share the same algorithm framework, the prediction approaches for MSH time series with missing values can be divided. Weerakody et al. [17] pointed out that although traditional machine learning and deep neural networks dominated the field of rule-based modeling, their inherent associations made it difficult to characterize the nonuniform sampling behaviors of time series effectively, especially under incomplete observation situations that were more limited. RNNs exhibited distinctive capabilities in temporal modeling, as they could adaptively capture missing patterns, time intervals, and multiscale temporal dependencies in nonuniformly sampled data. For the challenges of preprocessing irregular time series, two principal approaches had been considered: missing interpolation in the preprocessing stage and modification algorithms to directly handle missing values during model training. Chen et al. [18] proposed a Bayesian temporal factorization framework that implemented missing filling and multistep rolling prediction tasks in different manners. The graphical model could effectively perform probability prediction without requiring prior imputation of missing values. Therefore, in order to systematically reduce data errors, a feasible method for multisource time-series data preprocessing in DNs is to integrate statistical methods, traditional models, and intelligent networks for correlation analysis. For example, one may solve the synchronization and missing value filling of time-series data from different sources, and employ edge intelligence for distributed processing to reduce the impact of transmission delays by integrating heterogeneous time-series data such as meteorological/load data to build a unified analysis foundation. Simultaneously, jointly using statistical, optimization, and deep learning models may achieve error control of mechanism and data complementarity, comprehensively improving data quality to support subsequent applications.

### 3.2. Multisource Data Fusion

The dispatch center needs to dynamically configure the MSH state estimation cycle on account of the topological characteristics of the DNs and operational requirements. The goal is to construct a high-precision real-time grid operational representation based on redundant measurement data. Since the DN measurement system consists of multiple types of sensors, its measurement data flow exhibits MSH characteristics in the space–time dimension that forms a complex hybrid observation environment. Nevertheless, due to cost constraints and deployment conditions, existing MSH measurement devices have coverage limitations, which makes it difficult for a single data source to support the requirements of global DN observability [19].

The sampling characteristics of different types for measurement devices are various; thus, the fusion approaches of MSH data will directly determine the estimation accuracy. Lima et al. [20] integrated power transformer fault diagnosis with the decision-making process, involving both operational conditions and service life of the equipment. This provided guidance on decision-making methods through risk analysis and improved indicators derived from failure rates and Arrhenius theory. Liu et al. [21] developed a combined prediction model based on cross-entropy, and designed a variable time window mechanism to reduce potential losses caused by the delayed maintenance. Furthermore, an MSH fusion approach was designed by Ma et al. [22] for sensor error prediction utilizing kernel support vector regression and adaptive genetic approaches, which provided the solution to inadequate feature information and fusion behavior. Liu et al. [23] investigated a dual-channel CNN for fault recognition in DN systems, analyzed the fault mechanisms and waveform characteristics of different causes, and built a fully connected layer through multimodal MSH fusion to optimize classification.

**Figure 5:**
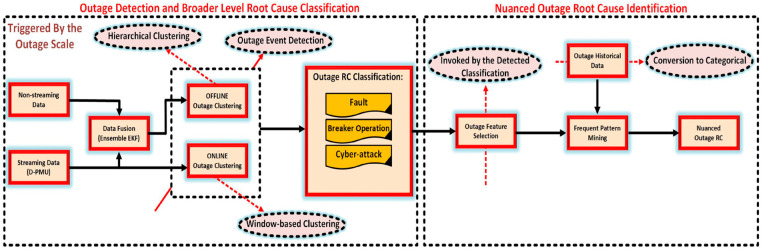
Overview of the outage root cause analysis for the distribution system in [29].

In addition, to address the problem of monitoring the DNs’ operation under an unknown distribution of sensor noise and measurement errors, Li et al. [24] provided a dynamic state estimation approach by using adaptive set membership filters. Zhu et al. [25] proposed a high-resolution state estimation framework for interval dynamic harmonics in DNs in line with MSH measurement fusion. It involved a selection strategy for the measurement location of harmonic electrical distance in DN power quality, an MSH data fusion with an exponential period, enhanced prediction correction, etc. In order to balance the information degradation induced by the errors from MSH measurement, quantization, and transmission, Seneviratne et al. [26] devised a statistical fusion approach that was applicable not only to sensor networks based on structural chains and trees, but also to unstructured bidirectional graph noise in harsh environments. On the basis of YOLOv5 architecture, Niu et al. [27] modified the GhostNet to reconstruct its original backbone network, which introduced a bidirectional feature pyramid structure for feature fusion and employed focal extended IoU to achieve the accuracy–speed balance. Yuan et al. [28] employed Bayesian networks to search for power outages in MSH distribution systems, and leveraged probability graphs to involve MSH evidence and the complex structure of DN systems. This could improve the efficiency of interruption location inference in high-dimensional spaces. As shown in Figure 5, Gholami et al. [29] designed an outage root cause analysis tool, which exploited the available data from many sensors within the DNs to (1) integrate via the MSH data fusion approach, (2) categorize events in a wider range by online- and offline-based hierarchical agglomerative clustering, and (3) take historical samples and frequent pattern growth mining approaches for subtle distinction detection.

To break through the contradiction between measurement configuration and estimation accuracy, the current research hotspots focus on advanced MSH information fusion. The primary objective in this field is to explore the multidimensional collaborative mechanism of steady-state data in SCADA systems, dynamic phasor data in D-PMU, and consumer-side data in AMI. By establishing a cross-modal association model, the granularity of state perception can be enhanced with reduced hardware investment costs. This not only helps to compensate for the observation blind spots of a single sensor, but also enhances the robustness against noise through MSH data complementarity.

### 3.3. Multisource Status Monitoring and Data Assessment

With the proliferation of renewable energy, more refined analysis is needed for the real-time status of MSH-DNs, which covers topology, basic parameters, and operating conditions. The high complexity of modern large-scale MSH distribution systems and frequent changes in network topology hinder the accurate identification of line parameters sensitive to extreme weather events. Inspired by intelligent networks, an increasing number of complex architectures have been proposed, such as the dynamic graph fusion networks by Wang et al. [30], to improve the joint identification of topology and line parameters.

Conventional machine learning classifiers are typically designed based on the assumption of class balance. When faced with imbalanced class distribution, the model training will be prone to representation bias towards rare categories. Specifically, in minority-class learning scenarios with insufficient samples, one may struggle to capture the distribution of key features that result in decision boundaries shifting towards dominant classes. This systematic bias not only induces rare class samples to be misjudged as majority classes, i.e., the “class cannibalization” phenomenon, but also leads to overall degradation of classification performance, which compromises requirements of precise recognition for key minority classes in real-world applications. To address these issues, reconstructing the sample distribution characteristics of the training set may alleviate the overfitting tendency towards specific categories, and simultaneously improve its generalization ability in mixed distribution scenarios. State-of-the-art approaches concentrate on developing adaptive sampling strategies and GAN-based techniques to achieve category-balanced MSH data reconstruction of DNs, while maintaining the original features [31]. Complementarily, a hybrid filtering framework was proposed in [32] to address the state estimation of power systems by considering the constraints of phasor measurement units. By combining extended Kalman filtering with statistical criteria, the measurement values were treated as inequality constraints of the DN state, as well as being understood as constrained optimization that maximizes probability. The problem of data imbalance can also be solved by artificially synthesizing new samples using the Synthetic Minority Oversampling Technique (SMOTE). It focuses on minority classes and finds their nearest neighbors for each minority-class sample. Then, a point on the connection between these samples and their neighbors is randomly selected to synthesize a new sample. This process is repeated until the number of minority and majority classes reaches a relative balance. Unlike simple random replication, SMOTE generates new samples through interpolation, increasing the diversity of minority-class samples and helping classifiers better learn decision boundaries, thereby improving their ability to recognize minority classes.

**Figure 6:**
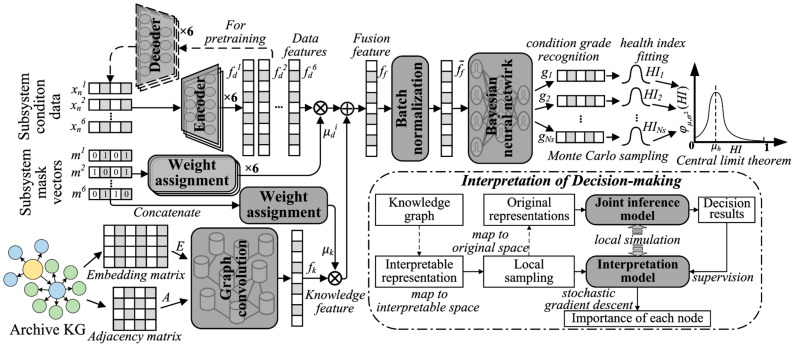
The interpretable joint inference method by subsystem measurements [35].

Further, heterogeneous data from PMUs and AMI devices employed in sensors has become prevalent in modern DNs [33]. In light of existing deficiencies in real-time monitoring and feedback capabilities, which hinder timely quantitative MSH assessment of potential early environmental risks, Sun et al. [34] summarized 10 environmental risks and classified them as related to the DN. They divided the monitoring samples into three dimensions and employed knowledge graphs to construct a potential external risk framework for generating dynamic visualizations. Recently, Tian et al. [35] optimized the interpretable joint reasoning approach by MSH subsystem measurement to address the manual summarization of rules, strong data dependency, lack of measurement analysis, rough information fusion, or inability to interpret evaluation results in previous approaches. A locally interpretable approach as illustrated in Figure 6 was designed to automatically extract MSH knowledge and data features through sparse autoencoders and a graph CNN, which reduced errors by considering extreme conditions. The expected status monitoring and data assessment will present the results of data monitoring, processing, and fusion in the form of state perception and full scenario evaluation, which also provide comprehensive visual information on key indicators. Combined with data analysis and extreme condition assessment, this will provide timely disaster warning information and optimize the operational efficiency of the power system as a scientific basis for decision-making.

**Figure 7:**
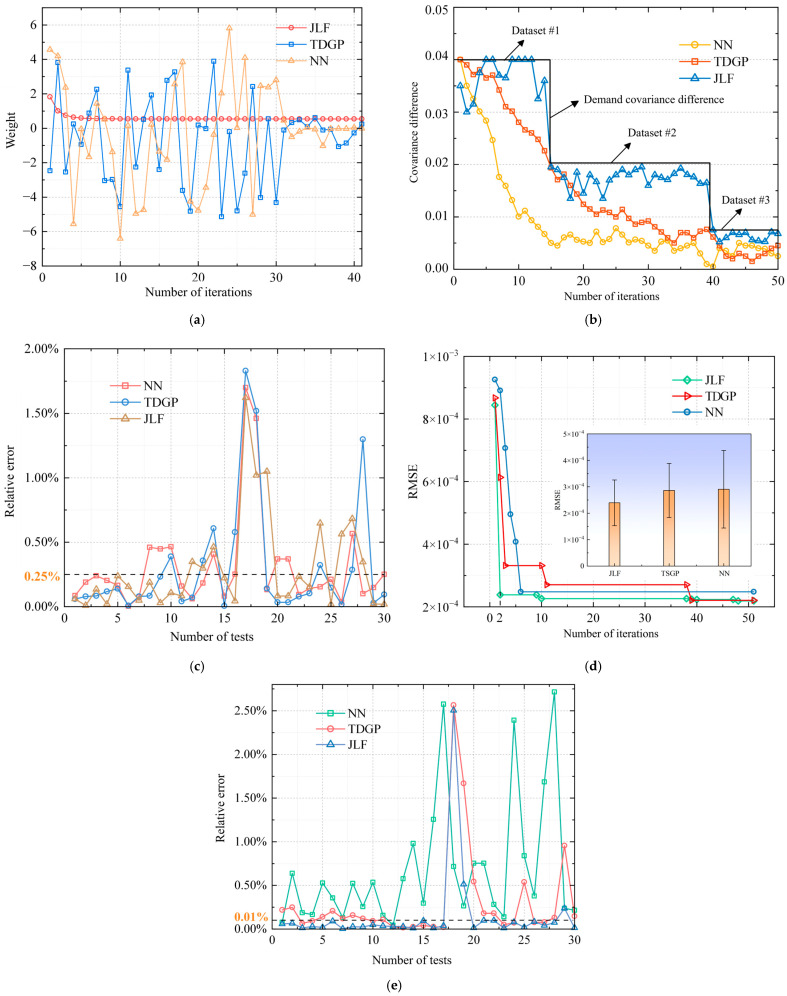
Examples of measured data fusion and processing. (**a**) Iterative curves of three algorithm weights in voltage data fusion. (**b**) Comparison of results of demand covariance of power under different data types and tracking covariance of three methods. (**c**) Comparison of results of relative errors in solar panel voltage. (**d**) Comparison of results of root mean square error of three methods for generating power. (**e**) Fusion error of power generation data.

Finally, by exploiting the MSH data preprocessing of the DNs, we conducted a preliminary study using the measured data from three photovoltaic platforms in a test region of Anhui Province, China. The data collected by the three mobile photovoltaic platforms mainly consists of two types, namely electrical data and meteorological data. Each day’s data can be recorded with a minimum interval of 15 min to centrally analyze the data changes within a day. This effectively combines information from different sensors or data sources with accuracy and reliability, and provides a basis for accurate DN state estimation, better decision-making, and management. Herein, the Time-domain Gaussian Process (TDGP), neural networks (NNs), and Joint Kalman filtering (JLF) are also tested for a comprehensive comparison, and the relevant results of the three methods based on a few examples from the datasets are compared in Figure 7.

It can be seen from Figure 7a that the JLF algorithm has almost no fluctuation in weights during iteration, which indicates that there is little conflict between data and good correlation. While using the TDGP model and NN algorithm, if there is a large fluctuation in the weights, there is a high degree of heterogeneity. By analyzing Figure 7b, it can be seen that all three data fusion methods can meet the given required covariance value. However, the fusion error of the NN algorithm is in a stable state and has not been adjusted according to the required accuracy; thus, the application effect is poor. Based on the TDGP model, there is also a high waste of accuracy, and the tracking fusion requires low error capability. The JLF algorithm may track the fusion error of requirements and have a good fusion effect. To evaluate the differences between different data sources, the absolute error between the demand covariance and tracking covariance of the JLF algorithm was further calculated. Among the datasets, datasets 1, 2, and 3 have probabilities of less than 0.1% of error values of 86.66%, 80%, and 90%, respectively. Based on the comparison of the fusion data of solar panel voltage and power generation, the JLF algorithm has the smallest relative error in the output value after fusion, which is smaller than the fusion errors of the other two methods. The average relative error of solar panel voltage and power generation is below 0.1%. Within 30 test cycles, the probabilities of voltage and power meeting the error are 83.33% and 80%, respectively. Compared with the other two algorithms, the JLF algorithm presented in Figure 7d converges the fastest and has the smallest RMSE value, which can improve the accuracy of fusion estimation. These preliminary research results enable readers to intuitively understand the performance of different methods and provide insights into the design and optimization of MSH sensors and processing approaches for practical applications in DNs. More detailed experiments and assessments will inspire discussions in future research.

## 4. Future Directions

**Figure 8:**
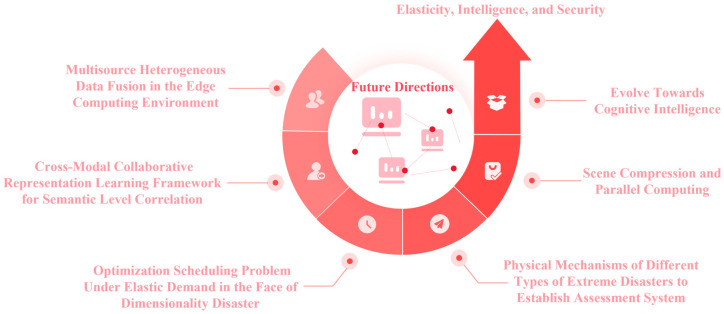
Future directions.

Rooted in the current progress of MSH power sensors, interdisciplinary integration, and practical development trends, this article provides the following aspects that demand further in-depth research to inspire potential researchers, as presented in Figure 8.

(1) Power systems and MSH sensors generate diverse unstructured and semi-structured data (e.g., signals, images, text, audio) that contains rich feature information. Potential research will concentrate on MSH data fusion approaches in the complex edge computing environment, establish a cross-modal data integration framework, and overcome the dependence of traditional monitoring systems on structured data. Priorities include unstructured MSH data feature extraction, spatiotemporal MSH correlation modeling, cognitive reasoning mechanisms, and multidimensional state perception establishment for DN power systems, which may provide the backbone for intelligent decision-making in both complex and extreme environmental scenarios.
(2) For the optimization scheduling issue under the elastic demand for power sensors, growing computational dimensionality, namely dimensionality disaster, is mainly induced by the large number of nodes and high complexity of grid structures. Since conventional algorithms struggle with this, the MSH computational complexity increases exponentially with the increase in variable dimensions that will put higher demands on the solving approaches.
(3) Computational complexity projected into high-dimensional MSH data space shows exponential growth. It is necessary to combine MSH scene compression and parallel computing techniques to transform global optimization problems into subproblem optimization. Simultaneously coupled with hardware parallel computing architectures, one may explore novel MSH computing coordination paradigms for breaking through the efficiency bottleneck, which will provide feasible real-time scheduling of large-scale power DNs.
(4) In terms of the physical mechanisms for distinct types of extreme disasters, it is necessary to establish a theoretical framework for assessing the specificity of DN disasters. For instance, earthquake disasters require the knowledge of the dynamic coupling effect between geological movement and power DN distributions, mountain fire scenarios require the analysis of the probability correlation between flame propagation and DN damage, and extreme temperature conditions also require the evaluation of DN thermal tolerance and load dynamic response. By constructing a refined MSH-based evaluation model, one may investigate the reinforcement of differentiated sensors and emergency dispatch strategies.
(5) Responding to high complexity and frequent occurrence of extreme disaster events in power DNs, future MSH fusion requires the construction of a cross-modal collaborative representation learning framework, which may integrate architectures such as deep residual networks, graph CNN, and Mamba [36,37,38], to achieve semantic level correlation between MSH equipment status, environmental perception, and DN operation control. By employing reinforcement learning to optimize resource scheduling strategies in disaster scenarios, one may enhance the rapid response and recovery capabilities of the DNs in extreme events, e.g., typhoons, rain, snow, and freezing, so as to form an intelligent integrated DN system with disaster resilience.
(6) To cope with the complexity of spatiotemporal evolution and disaster mutation in the power DNs, a hybrid architecture via an improved LSTM network and transformer may be considered to achieve dynamic environmental perception and real-time decision support for MSH data. Real-time collection of DN operation status and external disaster information through IoT technology is promising, and will be combined with large-scale MSH data analysis to dynamically regulate the power generation, transmission, and distribution strategies for guaranteeing continuous power supply for critical loads and improving the overall stability of DN sensor systems.
(7) Conforming to the development demands of future smart DNs, this research will also evolve towards cognitive intelligence, combining with small-sample unsupervised learning to build an MSH data fusion and assessment system with continuous evolution capabilities. By the visualization of the operation status of power DNs in extreme scenarios, one may optimize disaster prevention planning and response strategies for achieving rapid MSH fault location and triggering self-healing mechanisms. Eventually, this will ensure the safety and reliability of power sensors in complex disaster environments.

Looking ahead, there are numerous interdisciplinary fields worth exploring in addition to the aforementioned directions, which will continue to inspire the advancement of MSH sensor data processing research in DNs.

## 5. Conclusions

Addressing the demand for intelligent transformation of MSH sensors in power DNs, this article summarizes the technological development and typical approaches in terms of data processing. By comparative analysis of traditional engineering-oriented approaches and artificial intelligence technology, this article summarizes the technical characteristics and application scenarios into three major fields: MSH temporal preprocessing, data fusion, and state assessment.

Facing the development prospects of the convergence of new energy, the Internet, and technology, we have proposed several feasible directions, e.g., edge computing-based data fusion, cross-modal collaborative learning, optimal scheduling of disaster conditions, method evaluation by physical mechanism guidance, parallel computing, cognitive intelligence, etc. This article provides a reference for practitioners in power systems and DN sensor processing to grasp next-generation technology and optimization solutions.

## Figures and Tables

**Table 1:** Multisource data description for distribution networks.

Data type	Descriptions
Electrical data	(1) Basic electrical parameters Current: This includes phase currents, total currents, etc., used to monitor current changes in power sensors. Voltage: This includes phase voltage, line voltage, etc., used to monitor voltage fluctuations in power sensors. Power: This includes active power, reactive power, etc., reflecting the power output of the power sensors. (2) Power quality parameters Frequency: Monitoring the frequency changes of the power system to ensure frequency stability. Voltage deviation: Reflects the deviation between the voltage of the power system and the rated voltage. Three-phase imbalances: Monitoring the degree of imbalance in three-phase current or voltage. Harmonics: Monitoring the harmonic content in the power system to ensure the quality of electrical energy.
Meteorological data	Meteorological data carries a higher degree of spatiotemporal variability. The meteorological conditions vary greatly in different locations and time periods, and the drastic fluctuations in temperature may affect the measurement of humidity sensors, which may also lead to unreasonable anomalies in the MSH data. ★ Temperature: Monitoring the ambient temperature on site to evaluate the operating environment of the MSH equipment. ★ Humidity: Monitoring the humidity of the on-site environment. ★ Wind speed, wind direction, light intensity, atmospheric pressure: Used for photovoltaic modeling and prediction. ★ Other potential environmental data, e.g., smoke concentration, soil temperature, soil moisture, etc., are employed to monitor abnormal conditions in the on-site environment.
Hydrological data	This mainly includes precipitation, evaporation, water level, and flow rate, all of which are applicable for modeling and predicting hydroelectric power generation. ★ Precipitation denotes the vertical depth of liquid or solidified precipitation (melted) per unit area per unit time, which serves as the core indicator for hydrological measurement of total regional precipitation. ★ Evaporation quantifies the net water vapor flux from aquatic or terrestrial surfaces over a specific duration, which reflects the net loss of liquid water converted into water vapor at the evaporation surface. ★ The water level represents the elevation of the water surface relative to the standard baseline (e.g., the Yellow Sea’s average sea level), which reflects changes in water storage capacity. ★ Flow rate refers to the volumetric discharge through the cross-section of rivers, pipelines, and other water bodies per unit time, which directly determines water body transport capacity.
Other	(1) Switch and equipment status MSH switch status: Includes the closing and opening status of each switch, used to monitor changes in the switch status of the power sensors. MSH equipment operating conditions: Monitoring the operating status of the equipment, e.g., temperature, vibration, etc., to evaluate its health status. (2) Measurement and statistical data MSH electricity consumption: Recording the amount of electricity that passes through the line during a certain period. MSH statistical data: e.g., voltage qualification rate statistics, maximum current value statistics, etc. (3) Fault and alarm data MSH fault information: When a fault occurs in the MSH power system, the intelligent fusion terminal can collect real-time fault information, such as fault type, fault location, etc. MSH alarm information: When there is an abnormality or fault in the power system, the intelligent fusion terminal will upload the relevant alarm signal. (4) Control and regulate data MSH remote control data: The intelligent fusion terminal can receive control instructions from the higher-level system to obtain control remotely. MSH adjusting data: According to the actual demands of the MSH power system, the intelligent fusion terminal may output adjustment instructions, e.g., adjusting parameters such as voltage and current.

## Data Availability

The data presented in this study is available on request from the corresponding author.
